# Endometrial regenerative cells as a novel cell therapy attenuate experimental colitis in mice

**DOI:** 10.1186/s12967-014-0344-5

**Published:** 2014-12-05

**Authors:** Yongcheng Lv, Xiaoxi Xu, Bai Zhang, Guangying Zhou, Hongyue Li, Caigan Du, Hongqiu Han, Hao Wang

**Affiliations:** Department of General Surgery, Tianjin Medical University General Hospital, 154 Anshan Road, Heping District, Tianjin 300052 China; Tianjin General Surgery Institute, Tianjin, China; Department of Urologic Sciences, The University of British Columbia, Vancouver, BC Canada; Immunity and Infection Research Centre, Vancouver Coastal Health Research Institute, Vancouver, BC Canada

**Keywords:** Endometrial regenerative cells, Colitis, Mice

## Abstract

**Background:**

Endometrial regenerative cells (ERCs) are mesenchymal-like stem cells that can be non-invasively obtained from menstrual blood and are easily grown /generated at a large scale without tumorigenesis. We previously reported that ERCs exhibit unique immunoregulatory properties in vitro, however their immunosuppressive potential in protecting the colon from colitis has not been investigated. The present study was undertaken to determine the efficacy of ERCs in mediating immunomodulatory functions against colitis.

**Methods:**

Colitis was induced by 4% dextran-sulfate-sodium (DSS, in drinking water) in BALB/c mice for 7 days. ERCs were cultured from healthy female menstrual blood, and injected (1 million/mouse/day, i.v.) into mice on days 2, 5, and 8 following colitis induction. Colonic and splenic tissues were collected on day 14 post-DSS-induction. Clinical signs, disease activity index (DAI), pathological and immunohistological changes, cytokine profiles and cell populations were evaluated.

**Results:**

DSS-induced mice in untreated group developed severe colitis, characterized by body-weight loss, bloody stool, diarrhea, mucosal ulceration and colon shortening, as well as pathological changes of intra-colon cell infiltrations of neutrophils and Mac-1 positive cells. Notably, ERCs attenuated colitis with significantly reduced DAI, decreased levels of intra-colon IL-2 and TNF-α, but increased expressions of IL-4 and IL-10. Compared with those of untreated colitis mice, splenic dendritic cells isolated from ERC-treated mice exhibited significantly decreased MHC-II expression. ERC-treated mice also demonstrated much less CD3^+^CD25^+^ active T cell and CD3^+^CD8^+^ T cell population and significantly higher level of CD4^+^CD25^+^Foxp3^+^ Treg cells.

**Conclusions:**

This study demonstrated novel anti-inflammatory and immunosuppressive effects of ERCs in attenuating colitis in mice, and suggested that the unique features of ERCs make them a promising therapeutic tool for the treatment of ulcerative colitis.

## Background

Ulcerative colitis (UC) is a form of inflammatory bowel disease (IBD), characterized by the chronic, relapsing, non-specific inflammation of the colon and rectum. The inflammatory process in UC is confined to the mucosa. In contrast, Crohn’s disease (CD), another form of IBD, is characterized by segmental transmural mucosal inflammation and granulomatous changes, which affects any part of the gastrointestinal tract from the oral cavity to the perianal region [1]. Currently, UC has demonstrated high prevalence and incidence in the western countries, and these figures are rapidly rising in other parts of the world such as China during the past decades [2]. Despite extensive research, the definite cause of UC remains unclear. The general consensus appears to be that UC is related to individual genetic susceptibility, environmental risk factors or exposures, alterations of the gut microbiome and a deregulated immune system [3,4]. Conventional treatment for UC includes anti-inflammatory drugs, immunosuppressive agents, biological therapy and even colectomy [5]. However, these therapies often fail to obtain satisfactory results or even make the patients lose the best opportunity for surgical treatment. Additionally, it involves side effects resulting in many complications, such as osteoporosis, metabolism disorders, cushingoid features, infections, gastroduodenal mucosal injury and impaired wound healing [6–8]. In this view, searching for a novel therapeutic strategy to treat UC is needed [9].

In the recent years, stem cell-based therapy as a promising alternative solution draws considerable attention for UC treatment. As an attractive candidate in cell therapy, mesenchymal stem cells (MSCs), a group of self-renewing, pluripotent stromal cells that can differentiate into multiple lineages [10], can promote tissue repair and wound healing in UC [11,12]. In the meantime, MSCs possess immunomodulatory and anti-inflammatory properties. These cells can interfere with the function of certain immune cells such as DCs, natural killer cells (NKs), T cells and B cells by means of cell-to-cell interaction and soluble factor secretion [13–16]. Moreover, MSCs are relatively immune privileged and capable of evasion from allo-reactive immune response, due to the low expression of MHC-II and co-stimulatory molecules on the surface, which means these cells are transplantable between HLA-incompatible persons [14,17]. MSCs can be obtained from many different types of tissues in the body, such as bone marrow and adipose tissue. Among all cell types, bone marrow-derived MSCs are the best characterized population and have been proven to be effective in experimental UC models [12,18,19]. However, there exist some pitfalls of these sources, such as the invasive accessible method and related complications, less availability and limited proliferation capacity. Therefore, in addition to MSCs, searching for a new source of regenerative cells is warranted.

ERCs, a new mesenchymal-like cell population isolated from the menstrual blood, which present great regenerative potential during menstruation, satisfy the critical criteria of MSCs but overcome the hurdles of other conventional sources. Apart from the ease of abstraction, ERCs are marked by considerable expandability while maintaining karyotypic normality and differentiation capacity [20]. We and others have demonstrated that ERCs effectively prevent critical limb ischemia in mice, and most importantly, the therapeutic effect of ERC was observed despite the use of human cells in a xenogeneic animal model [21]. Furthermore, the efficacy of ERCs has also been tested in other experimental models of stroke [22] and heart failure [23]. However, whether ERCs could be feasible to simultaneously repair the damaged tissue and rectify the abnormal immune system of UC is unclear [20,21]. In this study, we investigated the anti-inflammatory and immunoregulatory properties of ERCs in DSS-induced colitis using systemic infusion of ERCs in a mouse model.

## Methods

### Animals

Eight-week-old male BALB/c mice (Aoyide Co., Tianjin, China) weighing 18 to 20 g were housed under conventional experimental environment with 12-hour light–dark cycle in the Animal Care Facility, Tianjin General Surgery Institute. The mice had a free access to commercial standard mouse diet and water. All experiments were conducted in accordance with the protocols approved by the Animal Care and Use Committee of Tianjin Medical University (China) according to the Chinese Council on Animal Care guidelines.

### Preparation of ERCs for in vivo treatment

According to Naoko Hida et al., ERCs were collected from approximately 10 ml of menstrual blood of eight women (20–30 years old) on the first day of menstruation after informed consent was obtained. The samples were suspended in Dulbecco’s modified Eagle’s medium (DMEM) high glucose supplemented with 10% fetal bovine serum (FBS), and split into two 10-cm dishes. The estimated adherent cell number at the start of culture was approximately 1 × 10^7^ [24].

### Experiment design

DSS-induced colitis model was established in mice according to Kihara, N et al. [25]. Twenty four BALB/c mice were randomly assigned to three groups, each consisting of eight animals: Group 1, naive mice drinking no-DSS water for 14 days, as normal control. Group 2, colitis in mice was induced by drinking 4% DSS for 7 days, followed by another 7 days consumption of normal water, as untreated colitis control. Group 3, mice with DSS-induced colitis were intravenously injected with ERCs (suspended in phosphate buffered saline (PBS), 1 × 10^6^/0.25 ml/mouse) on day 2, 4, 6, 8, as the study group. The body weight, stool consistency and characteristics of bloody stools of each mouse, as well as the DAI were evaluated during experiment. DAI was scored according to the following criteria (a) body weight loss: 0 (no change); 1 (1-5%); 2 (5-10%); 3 (10-20%); and 4 (>20%). (b) Stool consistency or diarrhea: 0 (normal); 1 (some soft); 2 (soft); 3 (unformed/mild diarrhea); and 4 (severe watery diarrhea). Hemoccult positivity and the presence of gross stool blood: 0 (negative fecal occult blood); 1 (negative/positive fecal occult blood); 2 (certain positive fecal occult blood); 3 (visible rectal bleeding); 4 (severe rectal bleeding). DAI is the sum score of body weight loss, stool consistency and gross bleeding, divided by 3. All mice were sacrificed on day14 post DSS-induction (as a uniform time point) for collection of spleen and colon samples and for the comparison of experimental data between ERC treated group and untreated group. The colon was excised from the ileocecal junction to the anus, and the length was measured. Followed by cutting longitudinally, the colon sections were washed in cold physiological saline solution and the colonic contents were removed. Half of the colon samples was fixed in 10% formalin and processed for paraffin embedding. The other part was stored frozen at −80°C.

### Histology

At necropsy, the colonic samples were fixed in 10% buffered formaldehyde, embedded in paraffin and sectioned into 5 μm for haematoxylin and eosin staining. As previously described, the microscopic sections were examined in a blinded fashion by a pathologist. Histological changes were scored according to the following criteria (a) inflammation severity: 0 (none), 1 (slight), 2 (moderate), 3 (severe); (b) depth of injury: 0 (none), 1 (mucosal), 2 (mucosal and submucosal), 3 (transmural); (c) crypt damage: 0 (none), 1 (basal 1/3 damage), 2 (basal 2/3 damage), 3 (crypt lost, only surface epithelium intact), 4 (entire crypt and epithelium lost); (d) percent involvement: 1 (1%-25%), 2 (26%-50%), 3 (51%-75%), 4 (76%-100%). Each parameter score was multiplied by a factor reflecting percent involvement of the intestinal wall and summed to obtain a histopathology score. The minimal score was 0 and the maximal score was 40 [26].

### Immunohistochemistry

To examine the protein expression levels of myeloperoxidase (MPO) and Mac-1 (CD11b) in the colon, the paraffin embedded colon specimens were cut into 5 μm, followed by deparaffined and rehydrated. Endogenous peroxides were blocked with 3%H_2_O_2_, and antigen retrieval was processed by heating in microwave. After enclosed by 5% bovine serum albumin (BSA), the specimens were stained according to the instructions of Strept Avidin-Biotin Complex (SABC) kit. The primary antigens were MPO goat anti-mouse antibody and Mac-1 goat anti-mouse antibody, and the secondary antigens were biotinylated goat anti-rabbit antigen. The negative control group was administered with PBS instead of primary antigen and stored frozen at 4°C overnight. The sections were graded by two blinded investigators to determine the immunoreactivity score according to the following criteria: (a) signal intensity: 0 (no staining), 1 (weak staining), 2 (moderate staining), or 3 (strong staining) and (b) percentage of positive cells in the tissue: 0 (0%), 1 (1%–10%), 2 (11%–25%), 3 (26%–50%), 4 (51%–75%), or 5 (76%–100%). Immunoreactivity score was calculated by multiplying the score for the percentage of positive cells by the intensity score (range of 0 to 15) [27].

### Real-time quantitative PCR

The gene transcriptional levels of IL-2, TNF-α, IL-4, IL-10 in colon tissues, and the gene transcriptional levels of IL-2, TNF-α in the spleen were determined by florescent real-time quantitative RT-PCR using real-time PCR instrument (MJ Research Inc., USA). The PCR primers were designed as follows: IL-2, upstream 5′-GGCATGTTCTGGATTTGACTC-3′, downstream 5′-CTCATCATCGAATTGGCACTC-3′; TNF-α, upstream 5′-CATCTTCTCAAAATTCGAGTGACAA-3′, downstream 5′-TGGGAGTAGACAAGGTACAACCC-3′; IL-4, upstream 5′-ACAGGAGAAGGGACGCCAT-3′, downstream 5′-GAAGCCCTACAGACGAGCTCA-3′; IL-10, upstream 5′- AGAAGCATGGCCCAGAAATCA-3, downstream 5′-GGCCTTGTAGACACCTTGGT-3′.

### Flow cytometry analysis

To test the cell count of CD3^+^CD25^+^ cells,CD3^+^CD8^+^ cells, CD4^+^CD25^+^ Foxp3^+^Tregs, CD11c^+^MHC-II^+^ DCs in the spleen, splenic single-cell suspensions were prepared with the final concentration of 1 × 10^7^/ml before immunofluorescent staining. Fluorescent antibodies, including CD3ε-FITC, CD8a-PerCP, CD4-PE, CD25-APC, Foxp3^+^-FITC, CD11c-PE, MHC-II-FITC (Miltenyi Biotec, Germany), were added to the suspensions respectively. After incubation for 10mins, cells were washed in PBS, followed by centrifuging for 10 mins and fixation in 1% polyoxymethylene for more than 2 hours. Cells were analyzed using flow cytometry.

### Statistical analysis

Statistical analysis was performed in SPSS version 17.0 software (SPSS Inc., Chicago, USA) and the experimental data were presented as mean ± SD. The DAI scores, flow cytometric data, histology/immunohistochemistry scores and RT-PCR results were compared among groups by using one-way ANOVA. Differences with *p* values less than 0.05 were considered significant.

## Results

### ERCs ameliorate the symptoms of DSS-induced colitis

**Figure 1:**
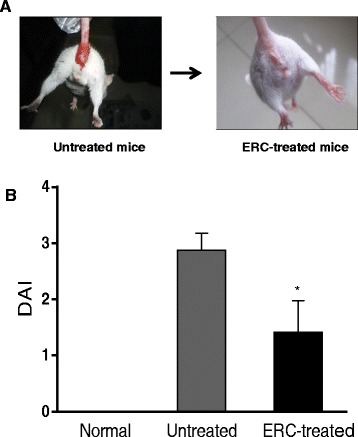
**ERCs attenuate the development of experimental colitis represented by DAI. A)** Representative photo showing bloody stool in untreated colitis mice as compared to ERC-treated colitis mice. **B)** DAI, scored daily to assess the colitis activity, in different groups (n = 8 mice per group). *p < 0.05, ERC-treated group vs. untreated group on day 14 post-colitis induction.

To determine the efficacy of ERCs in attenuation of colitis, we have evaluated clinical symptoms of DSS-induced colitis in mice. We found that untreated mice with colitis exhibited body weight loss, bloody loose stool and lethargy. In contrast, ERC-treated mice showed less body weight loss, firmer stool, as well as an increase in food and water consumption, indicating that the substantial wasting conditions caused by colitis were ameliorated by ERC treatment. Meanwhile, the incidence of bloody stool was clearly prevented in the ERC treated group as compared to that of the untreated group (Figure 1A). In addition, DAI, scored daily to assess the colitis activity, in ERC treated group was slightly higher than that of the normal group, however it was much lower than of the untreated group on day 14 post-colitis induction (*p < 0.05, Figure 1B).

### ERCs ameliorate histological changes in colon of the colitis mice

**Figure 2:**
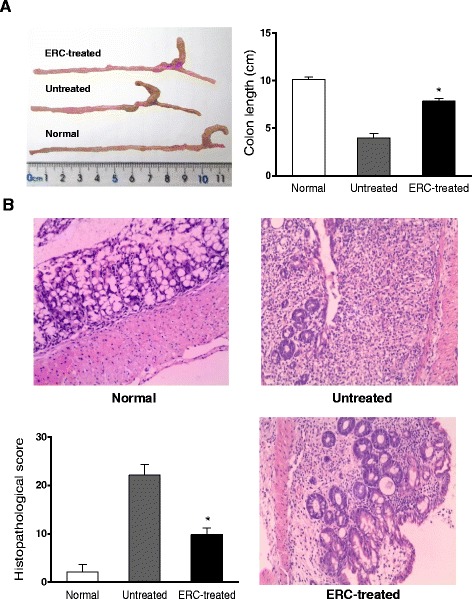
**ERCs attenuate the pathological changes of experimental colitis. A)** The length of mouse colon from normal, untreated and ERC-treated groups. *p < 0.05, ERC-treated group vs. untreated group. **B)** Representative photomicrographs (200×, haemotoxylin and eosin staining) of histological sections of colon from normal, untreated and ERC-treated groups. Overall histology scores in the normal, untreated and ERC-treated groups (n = 8 mice per group). *p < 0.05, ERC-treated group vs. untreated group.

Macroscopically, on day 14 post-colitis induction, mice in the untreated colitis group exhibited inflammation mainly in the distal colon with distal part constrictive and proximal part dilated. The lesions were characterized by mucosal hyperemia and ulceration. In the ERC treated group, obvious bowel dilation was not observed, and the length of colon was significantly longer than that of untreated colitis mice (*p < 0.05, Figure 2A). Meanwhile, mucosal hyperemia and edema were relieved, and the ulceration was almost healed (data not shown). Microscopically, in the colon collected from normal mice, intact epithelium and crypts were observed, with intestinal glands arranged in order, abundant goblet cells and few inflammatory cell infiltration. The pathology in untreated group showed severe inflammatory changes, characterized by damaged epithelium and crypts structure, glandular disorder, little goblet cell regeneration, and massive inflammatory cell infiltration into the mucosa and submucosa (Figure 2B). Whereas in the ERC treated group, mouse colon presented an improved structure of epithelium and crypts, with much less inflammatory cell infiltration but more regenerative goblet cells. In addition, the histopathological scores in ERC-treated group were significantly lower than that of untreated group (*p < 0.05), suggesting the less injury in the colon following ERC treatment.

### ERCs reduce intra-colon neutrophil and Mac-1 positive cell infiltration in the colitis mice

**Figure 3:**
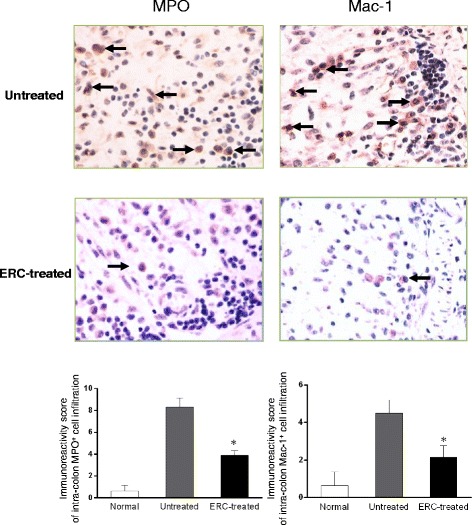
**ERCs reduce intra-colon neutrophil and Mac-1 positive cell infiltration in the colitis mice.** The levels of intra-colon MPO and Mac-1 positive cell infiltration in normal, untreated and ERC-treated groups. Magnification 400×. (n = 8 mice per group) *p < 0.05, ERC-treated group vs. untreated group.

To investigate the effects of ERCs on attenuation of inflammatory cell infiltration in the colitis mice, we have detected and compared intra-colon MPO (a biomarker for activated neutrophils) and Mac-1 positive cell infiltration in both untreated and ERC-treated colitis mice. As compared to untreated colitis mice, the levels of MPO positive neutrophils and Mac-1 positive cells, which include macrophages, NK cells and granulocytes were significantly reduced in the colon following ERCs treatment (*p < 0.05) (Figure 3).

### ERCs attenuate the transcriptional levels of cytokines in the colitis mice

**Figure 4:**
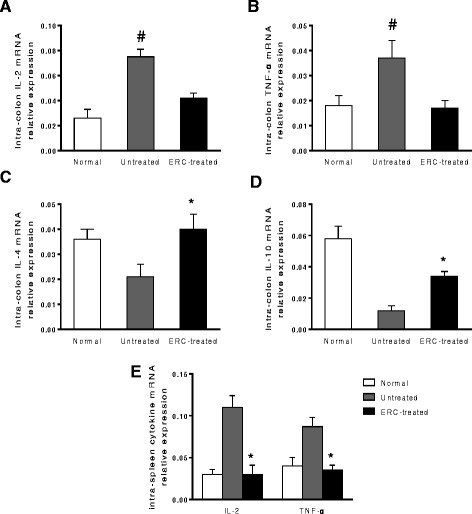
**Effects of ERCs on the regulation of IL-2, TNF-** **α** **, IL-4 and IL-10 expressions in mice.** The mRNA levels of IL-2, TNF-α, IL-4 and IL-10 were analyzed by real-time PCR. **A&B)** ERCs reduced IL-2 and TNF-α mRNA expression in colon tissues in DSS-induced colitis mice. (n = 8 mice per group) ^#^ *p* < 0.01, untreated group vs. normal mice and the ERC-treated group. **C&D)** ERCs increased IL-4 and IL-10 mRNA expression in colon tissues in DSS-induced colitis mice. **E)** ERCs reduced IL-2 and TNF-α mRNA expression in the spleen in DSS-induced colitis mice. (n = 8 mice per group) *p < 0.01, ERC-treated group vs. untreated group.

To determine whether ERC treatment would affect the transcriptional levels of cytokines, we have measured intra-colon expression of IL-2, IL-4, IL-10 and TNF-α by real-time PCR. As shown in Figure 4A-D, there were significant increases on IL-2 and TNF-α levels in colon of untreated colitis mice as compared with those of the normal mice and the ERC-treated colitis mice (^*#*^*p < 0.01*). In addition, both IL-4 and IL-10 were notably up-regulated in colon of the ERC treated group when compared with those of untreated group (*p < 0.01). In order to determine the systemic effects of ERC treatment on the transcriptional levels of cytokines, we have examined the levels of splenic IL-2 and TNF-α mRNA expression by real-time PCR. Following the transplantation of ERCs, the levels of these pro-inflammatory cytokines were significantly decreased (*p < 0.01), when compared with those of the untreated group (Figure 4E).

### ERC treatment modulate the number of immune cells in colitis mice

**Figure 5:**
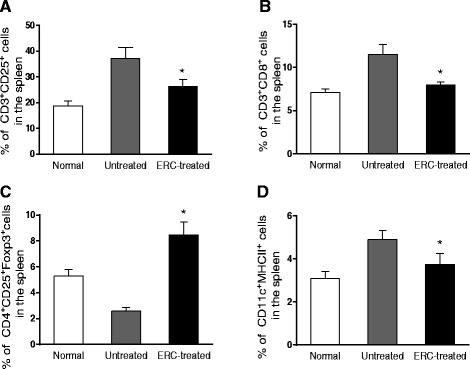
**ERCs modulate the levels of immune cells in the spleen of colitis mice.** The levels of immune cells were analyzed by flow cytometry in different groups. **A)** The level of CD3^+^CD25^+^ cells in the spleen from normal, untreated and ERC-treated groups. **B)** The level of CD3^+^CD8^+^ cells in the spleen from normal, untreated and ERC-treated groups. **C)** The level of CD4^+^CD25^+^ Foxp3^+^ Tregs in the spleen from normal, untreated and ERC-treated groups. **D)** The level of CD11c^+^MHC-II^+^ DCs in the spleen from normal, untreated and ERC-treated groups. (n = 8 mice per group) *p < 0.01, ERC-treated group vs. untreated group.

To further investigate the immunomodulatory function of ERCs in attenuation of colitis, the splenic immune cell populations were evaluated on day 14 post-colitis induction. We found that the populations of CD3^+^CD25^+^ active T cells, CD3^+^CD8^+^ T cells and CD11c^+^MHC-II^+^ DCs were significantly reduced by ERC treatment as compared to those of untreated colitis mice (*p < 0.01, Figure 5A, B, D). In addition, there was much higher level of CD4^+^CD25^+^Foxp3^+^ Tregs in ERC treated mice than that of untreated colitis mice and normal mice (*p < 0.01, Figure 5C).

## Discussion

Lately, cell therapy has been reported to be effective in the treatment of IBD, including UC and CD. MSCs, which can be obtained from many tissues, such as bone marrow and adipose tissue, are considered as promising candidates for cell therapy to treat UC. Currently, there are a few data on the therapeutic effects of ERCs, a novel population of stem cells acquired from menstrual blood. Other than the known advantages of MSCs, including proliferation and differentiation capacity, immunomodulatory property and immunoprivilege, ERCs possess additional outstanding merits, such as 1) abundant availability, 2) easy and painless extraction and isolation, 3) higher proliferation rate, 4) relative unlimited expandability without karyotypic or functional abnormality, 5) differentiation potential into more lineages [28]. In the current experiment, DSS–induced colitis model exhibited similar symptoms of UC patients, which include body weight loss, bloody stool and shortening of the colon. In the experimental colitis model, higher DAI scores were recorded which resembled the active stage. Under the microscopy, damaged epithelium and crypts were observed, as well as inflammatory cell infiltration which was confirmed by increased expression levels of MPO and Mac-1. Administration of ERCs ameliorated the symptoms, attenuated the microscopic pathological changes and the inflammatory cell infiltration, reflected by the reduced DAI scores and histological scores, respectively.

It is well known that UC is associated with abnormal immune system, where altered cell populations and cytokine secretion profiles play an important role [29]. In this study, to evaluate the related mechanism of ERCs, we have focused on their anti-inflammatory and immunomodulatory properties in attenuation of colitis. The results suggest that ERCs may have regulatory functions on the cell populations of splenic CD3^+^CD25^+^ active T cells, CD3^+^CD8^+^ T cells, CD4^+^CD25^+^ Foxp3^+^ Tregs and CD11c^+^MHC-II^+^ DCs in the colitis mice.

DCs are professional antigen-presenting cells which can take up antigens and present the processed antigens to other immune cells. The intestinal DCs have been investigated extensively. It was reported that UC is associated with the disruption of the delicate balance between the immunogenicity against invading pathogens and tolerance of the commensal microbiota, which is maintained by intestinal DCs at steady state [30]. In the current study, we have evaluated the number of splenic DCs distant from the intestine, and observed that the CD11c^+^MHC-II^+^ DCs increased significantly in the untreated colitis group. This was supported by previous reports, which stated that MSCs are capable of inhibiting the differentiation of monocytes into DCs [15,31–33]. This result suggests that ERCs probably reduce the cell number of DCs in the spleen, and exert immunomodulatory effects to control the development of colitis. Moreover, it was reported that splenic DCs may contribute to the systemic inflammation component, our finding further indicated the systemic therapeutic effects of ERCs [34].

As compared to untreated group, CD3^+^CD25^+^ active T cells were significantly downregulated after ERC treatment, indicating ERC might reduce T cell activation. To investigate the effects of ERCs on different T lymphocyte subsets, we have also measured the levels of CD4^+^CD25^+^ Foxp3^+^ Tregs and CD3^+^CD8^+^ T cells in different groups.

CD4^+^CD25^+^Foxp3^+^ Tregs account for 5%-10% of the CD4^+^ T cell panel in healthy human and mice, which are sufficient to play an important role in the maintenance of immune homeostasis and the limitation of autoimmune disease [35]. It has been shown that CD4^+^CD25^+^Foxp3^+^-deficient mice are hyperactive to intestinal commensal flora and would develop IBD triggered by infection. Transferring of CD4^+^CD25^+^Foxp3^+^ Tregs into immune deficient mice under aseptic conditions was capable of preventing or even reversing the development of IBD [36–38]. Similarly, our results demonstrated that CD4^+^CD25^+^Foxp3^+^ Tregs decreased significantly in the untreated colitis mice as compared to that of normal mice. However, the Treg population was markedly enhanced by intravenous injection of ERCs to the colitis mice, indicating that ERCs increase Tregs which may be associated with downregulation of autoimmune reaction and maintenance of the immune tolerance, thereby leading to the attenuation of colitis.

Furthermore, CD3^+^CD8^+^ T cells are capable to bind to the complex of MHC-Imolecules and antigens followed by the destruction of the target cells. The role of CD3^+^CD8^+^ T cells in the IBD remains unclear and controversial. It was claimed that cell-mediated cytotoxicity contributes to the exacerbation and perpetuation of active IBD [39]. According to some previous studies, antigen-specific CD8^+^ T cells are capable to induce intestinal inflammation triggered by epithelium-specific antigens which can be controlled by antigen-specific Tregs under physiological conditions [40]. The contribution of CD8^+^ T cell was confirmed by the in vivo monoclonal antibody depletion and cell- transfer experiments [41]. In our study, untreated colitis mice exhibited a high level of CD3^+^CD8^+^ T cells, in which was significantly down-regulated by the treatment of ERCs, suggesting that ERCs can down-regulate CD3^+^CD8^+^ T cell level, thereby ameliorating the cytotoxicity and correcting the dysfunctional immune system. This finding also indicated that, like MSCs, ERCs possess some immunomodulatory properties which could suppress the activation and proliferation of T cells and inhibit the differentiation of naïve CD8^+^ T cells into cytotoxic effector cells [42].

The data from the current study also suggest that ERCs could play a critical role in attenuating the pathogenesis of colitis through modulating the cytokine profile. Previous studies have revealed that the alterations of the cytokine production disrupt the balance of the immune system and have an impact on the pathogenesis of IBD [43–45]. Accumulating evidence has shown that MSCs exert immunomodulatory properties on IBD by changing the cytokine secretion profiles through paracrine [13,46]. Thus, the present study was undertaken to determine whether ERCs share the similar attribute of MSCs in amelioration of colitis partially through regulating cytokine profiles in this experimental model. It has been known that IL-2, the product of the activated T cells, is necessary for the proliferation and differentiation of naïve T cells into Th1 effector cells [47]. Our experiments demonstrated that high transcription level of IL-2 in colon tissues of untreated colitis mice were significantly down-regulated in ERC treated mice. This indicates that in the presence of ERCs, the secretion of Th1 (e.g. IL-2) cytokine was suppressed, which favors the shift of cytokine paradigm from a Th1 to a Th2 production, and facilitates the inhibition of the cell-mediated cytotoxity [48,49]. TNF-α, as a pro-inflammatory factor generated mainly by macrophages, plays a central role in the pathogenesis of UC [50]. The serum levels of TNF-α correlate with the clinical activity of UC. As indicated by previous reports, TNF-α blockers such as Infliximab are effective in the attenuation of inflammation, as well as the induction and maintenance of the long-term remission [51]. Our data showed that ERCs down-regulate the transcriptional level of TNF-α, suggesting its therapeutic benefits through anti-inflammation mechanism.

Moreover, our results also showed significantly increased transcriptional levels of IL-4 and IL-10 in the colon tissues of ERC-treated colitis mice compared to untreated colitis mice. As a result from the shift from Th1 to Th2 immune response, ERCs could promote the secretion of IL-4 followed by down-regulating inflammatory mediators such as TNF-α, IL-1 to exert anti-inflammatory and immunomodulatory effects. The increased level of IL-4 indicated a possible molecular therapeutic mechanism of ERCs on the colitis mice, which is consistent with previous findings that IL-4 has a protective function in intestinal tract of experimental colitis [52,53]. However, the role of IL-4 in IBD remains controversial, as to be either beneficial or detrimental in different experiment conditions [44,54,55]. The variations may result from the animal models, administration methods and differences in assay systems. Thus, further investigations are needed to clarify the role of IL-4 and to determine how ERCs exert therapeutic effects on IL-4 in the colitis. In addition, IL-10 is a potent anti-inflammatory cytokine that inhibits antigen presentation and the release of inflammatory factors [56]. IL-10-deficient mice are highly prone to chronic colitis and genomically-controlled human IL-10 expression rescued *Il10*^*−/−*^ mice from *Helicobacter*-induced colitis [45,57]. We found that ERC-treated mice had a dramatically increase in the transcriptional level of IL-10 compared to that of untreated mice. Therefore, we speculate that ERCs enhance the IL-10 level in the similar method reported by previous studies that MSCs can secrete IL-10 and promote the production of IL-10 by other antigen-presenting cells to exert anti-inflammatory and immunomodulatory effects [58,59].

Another intriguing feature of ERCs is their proliferative potential. According to previous reports, bone marrow-derived MSCs can effectively repair the injured tissues. Khalil et al. demonstrated that implanted MSCs are capable to induce the recovery of injured epithelium either by differentiation into the endothelial cells or improved angiogenesis [60]. Yujiro Hayashi et al. acclaimed that bone marrow-derived MSCs express VEGF and TGF-β1 both in vitro and after implantation to exert reparative effects in gastrointestinal wound healing [19]. Since ERCs are originated from endometrium which undergoes menstrual cycle, they are presumed to be capable of supporting angiogenesis and tissue remodeling. Evidence also shows that ERCs are of great therapeutic effects in the treatment of critical limb ischemia by stimulating angiogenesis [21]. Meanwhile, ERCs secrete matrix metalloproteases, which is important in tissue remodeling during the menstruation [28]. Thus, we assume that ERCs mediate the healing of tissue injury in this experimental colitis model through the similar tissue repair manner. The related study on ERCs is currently underway.

Interestingly, ERCs derived from human menstrual blood are tolerated and exhibit therapeutic effects on colitis in the immune-competent mice. The immunosuppressive effect of ERCs might have different mechanisms in xenogeneic settings as compared to those of allogeneic settings. However, we did find the immunoregulatory effects of ERCs in this experimental colitis xenogeneic model, implying that, in addition to their potential effects in angiogenesis, xenogeneic ERCs could play an important role in regulating immune responses against colitis in mice. This notion is supported by the reports that human umbilical cord blood MSCs reduced colitis in mice [61], and that human ERCs were able to suppress cell proliferation, IFN-γ and TNF-α production in a mouse mixed lymphocyte reaction [21]. This finding further supports the immune privilege property of ERCs and the possibility of utilization of allogeneic ERCs to treat UC in the clinical settings.

Although ERC-based cell therapy proves to be effective in attenuation of colitis, the precise mechanisms are not fully understood. For an instance, few data of the implanted sites of ERCs is recorded and the mechanism of homing is still elusive. Since ERCs are mesenchymal-like stem cells derived from the menstrual blood and express migration-related marker such as CD44 [62], and based on the data from our MSC-related publication [63], we speculate that ERCs could have the similar migration property as MSCs, and migrate to injured tissue and lymphoid organ in a similar mechanism in the colitis mice. Thus, the in-depth studies are warranted to clarify how ERCs are involved in the procedure.

## Conclusions

This study has demonstrated that ERC transplantation is effective in the treatment of DSS-induced colitis. After the intravenous administration, ERCs ameliorated the clinic symptoms and the histological changes in the colons of DSS-induced colitis in mice. ERCs reduced intra-colon inflammatory cell infiltration, and modulated the transcriptional levels of inflammatory cytokines, as well as the number of immune cells. Moreover, ERCs possess some advantages in comparison with MSCs from other sources including the ease of abstraction, considerable availability, obtaining the cells from a waste tissue and the expandability to great quantities without loss of differentiation ability or karyotypic abnormalities. In the light of their immunomodulatory and reparative capacity, ERCs could be a novel candidate for the treatment of IBD in clinic. This study serves as the basis for future studies, such as dose dependence study, toxicity and biocompatibility study. We suggest further clinical investigation of ERC is warranted.

## Abbreviations

CD: Crohn’s disease
DAI: Disease activity index
DC: Dendritic cell
DSS: Dextran surfate sodium
ERC: Endometrial regenerative cells
HLA: Human leukocyte antigen
IBD: Inflammatory bowel disease
IL: Interleukin
MHC: Major histocompatibility complex
MPO: Myeloperoxidase
MSC: Mesenchymal stem cells
SPF: Specific pathogen free
TGF-β1: Transforming growth factor-β1
Th: Helper T cell
TNF: Tumor necrosis factor
Treg: Regulatory T cell
UC: Ulcerative colitis
VEGF: Vascular endothelial growth factor

## References

[1] Dalal J, Gandy K, Domen J (2012). Role of mesenchymal stem cell therapy in Crohn’s disease. Pediatr Res.

[2] Ye L, Cao Q, Cheng J (2013). Review of inflammatory bowel disease in China. ScientificWorldJournal.

[3] Ponder A, Long MD (2013). A clinical review of recent findings in the epidemiology of inflammatory bowel disease. Clin Epidemiol.

[4] Baumgart DC, Carding SR (2007). Inflammatory bowel disease: cause and immunobiology. Lancet.

[5] Kornbluth A, Sachar DB, Practice Parameters Committee of the American College of G (2010). Ulcerative colitis practice guidelines in adults: American College Of Gastroenterology, Practice Parameters Committee. Am J Gastroenterol.

[6] Toruner M, Loftus EV, Harmsen WS, Zinsmeister AR, Orenstein R, Sandborn WJ, Colombel JF, Egan LJ (2008). Risk factors for opportunistic infections in patients with inflammatory bowel disease. Gastroenterology.

[7] Naganuma M, Kunisaki R, Yoshimura N, Takeuchi Y, Watanabe M (2013). A prospective analysis of the incidence of and risk factors for opportunistic infections in patients with inflammatory bowel disease. J Gastroenterol.

[8] Sternthal MB, Murphy SJ, George J, Kornbluth A, Lichtiger S, Present DH (2008). Adverse events associated with the use of cyclosporine in patients with inflammatory bowel disease. Am J Gastroenterol.

[9] Singh UP, Singh NP, Singh B, Mishra MK, Nagarkatti M, Nagarkatti PS, Singh SR (2010). Stem cells as potential therapeutic targets for inflammatory bowel disease. Front Biosci (Schol Ed).

[10] Larsen S, Lewis ID (2011). Potential therapeutic applications of mesenchymal stromal cells. Pathology.

[11] Luo Q, Zhang C, Song G (2012). Research progresses of paracrine effect of bone marrow derived mesenchymal stem cells on wound healing. Sheng Wu Yi Xue Gong Cheng Xue Za Zhi.

[12] Xu X, Zhu F, Zhang M, Zeng D, Luo D, Liu G, Cui W, Wang S, Guo W, Xing W, Liang H, Li L, Fu X, Jiang J, Huang H (2013). Stromal cell-derived factor-1 enhances wound healing through recruiting bone marrow-derived mesenchymal stem cells to the wound area and promoting neovascularization. Cells Tissues Organs.

[13] He XW, He XS, Lian L, Wu XJ, Lan P (2012). Systemic infusion of bone marrow-derived mesenchymal stem cells for treatment of experimental colitis in mice. Dig Dis Sci.

[14] De Miguel MP, Fuentes-Julian S, Blazquez-Martinez A, Pascual CY, Aller MA, Arias J, Arnalich-Montiel F (2012). Immunosuppressive properties of mesenchymal stem cells: advances and applications. Curr Mol Med.

[15] Molendijk I, Duijvestein M, van der Meulen-de Jong AE, van Deen WK, Swets M, Hommes DW, Verspaget HW (2012). Immunomodulatory effects of mesenchymal stromal cells in Crohn’s disease. J Allergy (Cairo).

[16] Soleymaninejadian E, Pramanik K, Samadian E (2012). Immunomodulatory properties of mesenchymal stem cells: cytokines and factors. Am J Reprod Immunol.

[17] Le Blanc K, Tammik C, Rosendahl K, Zetterberg E, Ringden O (2003). HLA expression and immunologic properties of differentiated and undifferentiated mesenchymal stem cells. Exp Hematol.

[18] Li M, Ikehara S (2013). Bone-marrow-derived mesenchymal stem cells for organ repair. Stem Cells Int.

[19] Hayashi Y, Tsuji S, Tsujii M, Nishida T, Ishii S, Iijima H, Nakamura T, Eguchi H, Miyoshi E, Hayashi N, Kawano S (2008). Topical implantation of mesenchymal stem cells has beneficial effects on healing of experimental colitis in rats. J Pharmacol Exp Ther.

[20] Meng X, Ichim TE, Zhong J, Rogers A, Yin Z, Jackson J, Wang H, Ge W, Bogin V, Chan KW, Thebaud B, Riordan NH (2007). Endometrial regenerative cells: a novel stem cell population. J Transl Med.

[21] Murphy MP, Wang H, Patel AN, Kambhampati S, Angle N, Chan K, Marleau AM, Pyszniak A, Carrier E, Ichim TE, Riordan NH (2008). Allogeneic endometrial regenerative cells: an “Off the shelf solution” for critical limb ischemia?. J Transl Med.

[22] Borlongan CV, Kaneko Y, Maki M, Yu SJ, Ali M, Allickson JG, Sanberg CD, Kuzmin-Nichols N, Sanberg PR (2010). Menstrual blood cells display stem cell-like phenotypic markers and exert neuroprotection following transplantation in experimental stroke. Stem Cells Dev.

[23] Bockeria L, Bogin V, Bockeria O, Le T, Alekyan B, Woods EJ, Brown AA, Ichim TE, Patel AN (2013). Endometrial regenerative cells for treatment of heart failure: a new stem cell enters the clinic. J Transl Med.

[24] Hida N, Nishiyama N, Miyoshi S, Kira S, Segawa K, Uyama T, Mori T, Miyado K, Ikegami Y, Cui C, Kiyono T, Kyo S, Shimizu T, Okano T, Sakamoto M, Ogawa S, Umezawa A (2008). Novel cardiac precursor-like cells from human menstrual blood-derived mesenchymal cells. Stem Cells.

[25] Kihara N, de la Fuente SG, Fujino K, Takahashi T, Pappas TN, Mantyh CR (2003). Vanilloid receptor-1 containing primary sensory neurones mediate dextran sulphate sodium induced colitis in rats. Gut.

[26] Chidlow JH, Langston W, Greer JJ, Ostanin D, Abdelbaqi M, Houghton J, Senthilkumar A, Shukla D, Mazar AP, Grisham MB, Kevil CG (2006). Differential angiogenic regulation of experimental colitis. Am J Pathol.

[27] Liang Y, Hou X, Cui Q, Kang TB, Fu JH, Zhang LJ, Luo RZ, He JH, Zeng YX, Yang HX (2012). Skp2 expression unfavorably impacts survival in resectable esophageal squamous cell carcinoma. J Transl Med.

[28] Zhong Z, Patel AN, Ichim TE, Riordan NH, Wang H, Min WP, Woods EJ, Reid M, Mansilla E, Marin GH, Drago H, Murphy MP, Minev B (2009). Feasibility investigation of allogeneic endometrial regenerative cells. J Transl Med.

[29] Neurath MF (2014). Cytokines in inflammatory bowel disease. Nat Rev Immunol.

[30] Al-Hassi HO, Mann ER, Sanchez B, English NR, Peake ST, Landy J, Man R, Urdaci M, Hart AL, Fernandez-Salazar L, Lee GH, Garrote JA, Arranz E, Margolles A, Stagg AJ, Knight SC, Bernardo D (2014). Altered human gut dendritic cell properties in ulcerative colitis are reversed by Lactobacillus plantarum extracellular encrypted peptide STp. Mol Nutr Food Res.

[31] Kim MG, Kim SH, Noh H, Ko YS, Lee HY, Jo SK, Cho WY, Kim HK (2013). CD11c(+) cells partially mediate the renoprotective effect induced by bone marrow-derived mesenchymal stem cells. PLoS One.

[32] Choi YS, Jeong JA, Lim DS (2012). Mesenchymal stem cell-mediated immature dendritic cells induce regulatory T cell-based immunosuppressive effect. Immunol Invest.

[33] Chiesa S, Morbelli S, Morando S, Massollo M, Marini C, Bertoni A, Frassoni F, Bartolome ST, Sambuceti G, Traggiai E, Uccelli A (2011). Mesenchymal stem cells impair in vivo T-cell priming by dendritic cells. Proc Natl Acad Sci U S A.

[34] Drakes ML, Blanchard TG, Czinn SJ (2005). Colon lamina propria dendritic cells induce a proinflammatory cytokine response in lamina propria T cells in the SCID mouse model of colitis. J Leukoc Biol.

[35] Himmel ME, Yao Y, Orban PC, Steiner TS, Levings MK (2012). Regulatory T-cell therapy for inflammatory bowel disease: more questions than answers. Immunology.

[36] Coombes JL, Robinson NJ, Maloy KJ, Uhlig HH, Powrie F (2005). Regulatory T cells and intestinal homeostasis. Immunol Rev.

[37] Maloy KJ, Antonelli LR, Lefevre M, Powrie F (2005). Cure of innate intestinal immune pathology by CD4 + CD25+ regulatory T cells. Immunol Lett.

[38] Himmel ME, Hardenberg G, Piccirillo CA, Steiner TS, Levings MK (2008). The role of T-regulatory cells and Toll-like receptors in the pathogenesis of human inflammatory bowel disease. Immunology.

[39] Muller S, Lory J, Corazza N, Griffiths GM, Z’Graggen K, Mazzucchelli L, Kappeler A, Mueller C (1998). Activated CD4+ and CD8+ cytotoxic cells are present in increased numbers in the intestinal mucosa from patients with active inflammatory bowel disease. Am J Pathol.

[40] Westendorf AM, Fleissner D, Deppenmeier S, Gruber AD, Bruder D, Hansen W, Liblau R, Buer J (2006). Autoimmune-mediated intestinal inflammation-impact and regulation of antigen-specific CD8+ T cells. Gastroenterology.

[41] Nancey S, Holvoet S, Graber I, Joubert G, Philippe D, Martin S, Nicolas JF, Desreumaux P, Flourie B, Kaiserlian D (2006). CD8+ cytotoxic T cells induce relapsing colitis in normal mice. Gastroenterology.

[42] Lotfinegad P, Shamsasenjan K, Movassaghpour A, Majidi J, Baradaran B (2014). Immunomodulatory nature and site specific affinity of mesenchymal stem cells: a hope in cell therapy. Adv Pharm Bull.

[43] Torres MI, Rios A (2008). Current view of the immunopathogenesis in inflammatory bowel disease and its implications for therapy. World J Gastroenterol.

[44] Muzes G, Molnar B, Tulassay Z, Sipos F (2012). Changes of the cytokine profile in inflammatory bowel diseases. World J Gastroenterol.

[45] Sanchez-Munoz F, Dominguez-Lopez A, Yamamoto-Furusho JK (2008). Role of cytokines in inflammatory bowel disease. World J Gastroenterol.

[46] Doorn J, Moll G, Le Blanc K, van Blitterswijk C, de Boer J (2012). Therapeutic applications of mesenchymal stromal cells: paracrine effects and potential improvements. Tissue Eng Part B Rev.

[47] Smith KA (1988). Interleukin-2: inception, impact, and implications. Science.

[48] Parekkadan B, Milwid JM (2010). Mesenchymal stem cells as therapeutics. Annu Rev Biomed Eng.

[49] Mok PL, Leong CF, Cheong SK (2013). Cellular mechanisms of emerging applications of mesenchymal stem cells. Malays J Pathol.

[50] Apostolaki M, Armaka M, Victoratos P, Kollias G (2010). Cellular mechanisms of TNF function in models of inflammation and autoimmunity. Curr Dir Autoimmun.

[51] Lv R, Qiao W, Wu Z, Wang Y, Dai S, Liu Q, Zheng X (2014). Tumor necrosis factor alpha blocking agents as treatment for ulcerative colitis intolerant or refractory to conventional medical therapy: a meta-analysis. PLoS One.

[52] Chen LL, Wang XH, Cui Y, Lian GH, Zhang J, Ouyang CH, Lu FG (2009). Therapeutic effects of four strains of probiotics on experimental colitis in mice. World J Gastroenterol.

[53] Macdonald TT (1998). Viral vectors expressing immunoregulatory cytokines to treat inflammatory bowel disease. Gut.

[54] Xiong J, Lin YH, Bi LH, Wang JD, Bai Y, Liu SD (2013). Effects of interleukin-4 or interleukin-10 gene therapy on trinitrobenzenesulfonic acid-induced murine colitis. BMC Gastroenterol.

[55] Iijima H, Takahashi I, Kishi D, Kim JK, Kawano S, Hori M, Kiyono H (1999). Alteration of interleukin 4 production results in the inhibition of T helper type 2 cell-dominated inflammatory bowel disease in T cell receptor alpha chain-deficient mice. J Exp Med.

[56] Iyer SS, Cheng G (2012). Role of interleukin 10 transcriptional regulation in inflammation and autoimmune disease. Crit Rev Immunol.

[57] Ranatunga DC, Ramakrishnan A, Uprety P, Wang F, Zhang H, Margolick JB, Brayton C, Bream JH (2012). A protective role for human IL-10-expressing CD4+ T cells in colitis. J Immunol.

[58] Dorronsoro A, Fernandez-Rueda J, Fechter K, Ferrin I, Salcedo JM, Jakobsson E, Trigueros C (2013). Human Mesenchymal Stromal Cell-Mediated Immunoregulation: Mechanisms of Action and Clinical Applications. Bone Marrow Res.

[59] Shi Y, Su J, Roberts AI, Shou P, Rabson AB, Ren G (2012). How mesenchymal stem cells interact with tissue immune responses. Trends Immunol.

[60] Khalil PN, Weiler V, Nelson PJ, Khalil MN, Moosmann S, Mutschler WE, Siebeck M, Huss R (2007). Nonmyeloablative stem cell therapy enhances microcirculation and tissue regeneration in murine inflammatory bowel disease. Gastroenterology.

[61] Kim HS, Shin TH, Lee BC, Yu KR, Seo Y, Lee S, Seo MS, Hong IS, Choi SW, Seo KW, Nunez G, Park JH, Kang KS (2013). Human umbilical cord blood mesenchymal stem cells reduce colitis in mice by activating NOD2 signaling to COX2. Gastroenterology.

[62] Sackstein R, Merzaban JS, Cain DW, Dagia NM, Spencer JA, Lin CP, Wohlgemuth R (2008). Ex vivo glycan engineering of CD44 programs human multipotent mesenchymal stromal cell trafficking to bone. Nat Med.

[63] Ge W, Jiang J, Baroja ML, Arp J, Zassoko R, Liu W, Bartholomew A, Garcia B, Wang H (2009). Infusion of mesenchymal stem cells and rapamycin synergize to attenuate alloimmune responses and promote cardiac allograft tolerance. Am J Transplant.

